# Immunotherapies inducing immunogenic cell death in cancer: insight of the innate immune system

**DOI:** 10.3389/fimmu.2023.1294434

**Published:** 2023-11-23

**Authors:** Kenny Misael Calvillo-Rodríguez, Helen Yarimet Lorenzo-Anota, Cristina Rodríguez-Padilla, Ana Carolina Martínez-Torres, Daniel Scott-Algara

**Affiliations:** ^1^ Laboratorio de Inmunología y Virología, Facultad de Ciencias Biológicas, Universidad Autónoma de Nuevo León, San Nicolás de los Garza, NL, Mexico; ^2^ The Institute for Obesity Research, Tecnológico de Monterrey, Monterrey, NL, Mexico; ^3^ Département d'Immunologie, Unité de Biologie Cellulaire des Lymphocytes, Pasteur Institute, Paris, France

**Keywords:** onco-immunotherapy, immunogenic cell death, innate immune system, monoclonal antibodies, cytokines, oncolytic virus, cellular therapies, immunomodulators

## Abstract

Cancer immunotherapies include monoclonal antibodies, cytokines, oncolytic viruses, cellular therapies, and other biological and synthetic immunomodulators. These are traditionally studied for their effect on the immune system’s role in eliminating cancer cells. However, some of these therapies have the unique ability to directly induce cytotoxicity in cancer cells by inducing immunogenic cell death (ICD). Unlike general immune stimulation, ICD triggers specific therapy-induced cell death pathways, based on the release of damage-associated molecular patterns (DAMPs) from dying tumour cells. These activate innate pattern recognition receptors (PRRs) and subsequent adaptive immune responses, offering the promise of sustained anticancer drug efficacy and durable antitumour immune memory. Exploring how onco-immunotherapies can trigger ICD, enhances our understanding of their mechanisms and potential for combination strategies. This review explores the complexities of these immunotherapeutic approaches that induce ICD, highlighting their implications for the innate immune system, addressing challenges in cancer treatment, and emphasising the pivotal role of ICD in contemporary cancer research.

## 1 Introduction

Cancer immunotherapy aims to harness the patient’s own immune system to target and eliminate malignant cells. In 2013, cancer immunotherapy was named as “Breakthrough of the Year” by the journal Science for its promising potential in the field of oncology (1). Immunotherapy is often combined with other cancer treatments such as chemotherapy, surgery, radiotherapy, and targeted therapies, which aim to eliminate cancer cells by killing them. The combination of immune system activation with cancer cell-killing not only triggers the immune response but also precisely targets and eliminates cancer cells. This fusion demonstrates a markedly enhanced antitumor effect. In this way, cancer cells are eliminated by direct killing, while at the same time the immune system is activated.

Recently, ICD has been described as a promising form of therapy-induced anti-tumour immune system activation, and it is now considered to play a central role in various cancer treatment modalities. Although ICD targets cancer cells and mediates tumour‐specific immune responses, it occurs in the precise context of cell death induced by a specific therapy, making it conceptually distinct from cases of immune stimulation or inflammatory responses that do not rely on a therapy capable of inducing a specific modality of cell death (2). Thus, although ICD could be considered a form of immunotherapy, it has not been classified as such because it depends on a specific treatment that must be able to induce a specific cell death pathway that ends with a dying cancer cell that has sufficient antigenicity and adjuvanticity able to activate the immune system. ICD is characterized by the exposure and release of DAMPs from dying tumour cells that confer adjuvanticity. DAMPs are recognised by innate PRRs, resulting in the activation of innate cells and the subsequent activation of adaptive cells that mediate tumour‐specific immune responses. ICD is a promising strategy because it induces long‐term efficacy of anticancer drugs through the combination of the direct cancer cell killing and the activation of the antitumour immune system, leading to an anti-tumour immunological memory (3).

Some chemotherapies, radiotherapies, and targeted therapies have been shown to induce immunogenic cell death (4–6). Such ICD inductors have been successfully combined with different types of immunotherapies to promote better outcomes (7, 8). However, some immunotherapies, in parallel with their immunomodulatory effect that targets the immune system, can also be directly cytotoxic to the cancer cell and induce ICD. This article will review these specific types of immunotherapies, with an additional focus on their impact on the innate immune system.

## 2 Immunotherapies for cancer treatment

Oncology met immunology in 1891 when William B. Coley noticed that cancer patients who got infections after surgery seemed to do better than those who didn’t. So he tried immunotherapy for cancer by using erysipelas on a patient with inoperable sarcoma (9). He then created a filtered mixture of bacterial lysates called “Coley’s Toxins” to treat tumours. His first patient, John Ficken, with a large inoperable tumour (probably a malignant sarcoma) had a complete remission that lasted until his death from a heart attack 26 years later. Coley’s toxins may have stimulated the immune system to attack cancer cells. Thereafter, clinical interest in onco-immunotherapy waned, with research focusing on radiotherapy and chemotherapy (10–12), until 2013, when immunotherapy of cancer was named “Breakthrough of the Year” by Science (1).

Cancer is characterised by a number of features including activation of oncogenes, inactivation of tumour suppressor genes, resistance to cell death, angiogenesis, maintenance of proliferative signalling, immune suppression and avoidance of immune destruction (13). Even during cancer immunosurveillance, the most immunoevasive or highly mutagenic cancer cells may acquire the ability to evade immunosurveillance and thus generate a clinically relevant tumour. In this sense, cancer cells in an established tumour can evade anti-tumour immunity. In addition to the immunosuppressive microenvironment within the tumour, cancer cells can use several mechanisms for immunoevasion, which include (1): reducing their immunogenicity through the downregulation of tumour-associated antigens (TAAs) and major histocompatibility complex (MHC) class I expression (2), inducing tolerance by suppressing T cells (CD4+ and CD8+) through the promotion of immunosuppressive cytokines (e.g. IL-10 or TGFβ) or immune-checkpoints (e.g. regulated cell death 1, regulated cell death-ligand, cytotoxic T lymphocyte-associated protein-4), and (3) avoiding the immune cell-mediated lysis by overriding cell death pathways (14, 15), among others.

Pharmacological induction of cell death is the basis of almost all non-invasive cancer therapies. One of the major challenges in cancer treatment is to restore an anti-tumour immune response. In this sense, immunotherapies have transformed cancer treatment in recent years and have revitalised the field of tumour immunology (16). Cancer immunotherapy aims to stimulate the immune system in a controlled manner to eliminate cancer cells and prevent uncontrolled autoimmune inflammatory responses that lead to contraindications and therapeutic limitations (17). The main goals are to increase the quality or quantity of immune cells (especially effector cells), to generate tumour antigens and to eliminate mechanisms associated with immunosuppression, while minimising off-target effects. In addition, immunotherapies seek to induce long-lasting and durable responses in several cancer subsets, including solid and haematological malignancies. In this sense, several types of cancer immunotherapies have been developed with the aim of promoting cancer remission (18). These onco-immunotherapies are very vast and include (1) monoclonal antibodies (2), cytokines (3), oncolytic viruses (4), cellular therapies (5), and other biological and synthetic immunomodulators.

Activation of the anti-tumour immune system requires treatment strategies that can overcome the physiological barriers that control immune responses against tumour cells. Accordingly, immunotherapy uses strategies that target specific immunoregulatory processes to enhance anti-tumour immunity. However, cancer immunoediting can occur in response to immunotherapy as well as during tumour development. In this sense, immunotherapy may induce secondary (acquired) resistance that manifests as a clinical response followed by cancer progression (secondary escape) (19).

In general, the resistance of most cancers to immunotherapies and the lack of anti-tumour memory underline the need to overcome the immunosuppressive microenvironment, improve the immunogenicity of tumour cells and promote the induction of anti-tumour memory, rather than focusing only on stimulating broad and untargeted immune responses (20). In this sense, a novel strategy to induce immune system activation, antitumour memory and tumour microenvironment remodelling is the induction of a specific form of cell death called immunogenic cell death.

## 3 Immunogenic cell death in cancer

The immunogenicity of cancer cells has been identified as an essential factor in the development of anti-cancer therapies. Therefore, new research has focused on understanding the immunobiology of tumours in order to overcome the immunosuppressive function of the tumour microenvironment (TME) and increase the immunogenicity of cancer cells (21). In this sense, ICD is characterised by the increased immunogenicity of the cells (acting as a tumour vaccine) and the release of DAMPs, leading to the generation of immunological memory (21).

ICD is a type of cell death that can promote the antitumour immune response and induce immunological memory against endogenous (cellular) or exogenous (viral) antigens. The ability of ICD to stimulate adaptive immunity comprises two main parameters: antigenicity and adjuvanticity. Antigenicity is the ability of a molecule, such as a protein, to be recognised as an antigen and to promote an inflammatory response. This is provided by the production and presentation of antigens in the context of central tolerance in a given host that do not lead to clonal deletion, indicating that the host has naive T cell clones that can recognise such antigens. Adjuvanticity is mainly provided by the release or exposure of danger signals such as DAMPs due to cell damage or stress, and by pathogen-associated molecular patterns (PAMPs) in pathogen-derived ICD, which promote the recruitment and maturation of dendritic cells (DCs). These molecules have non-immunological effects within the cell, but, their exposure on the cell surface or their release into the extracellular space due to cellular stress allow their binding to receptors in immune cells (14, 21).

Cancer research has undergone a significant paradigm shift in recent years, with increasing emphasis on the importance of ICD in the context of cancer therapy. Both preclinical and clinical data have converged to support the notion that the way cancer cells undergo cell death in response to treatment carries is more important for long-term disease outcome than the proportion of cells that die. Given the challenge posed by the inability of current cancer therapies to achieve the utopian goal of eradicating 100% of cancer cells, there is a growing consensus among scientists for a strategic shift in focus. Rather than seeking cell death in isolation, the forefront of cancer research is now centred on the development of innovative combination therapeutic regimens designed to stimulate the antitumour immune system and induce cancer cell death (22–24).

Therefore, the use of immunotherapies that can both stimulate the immune system and induce ICD is of great interest because they can enhance the immune system’s ability to fight cancer while killing cancer cells. In the next section, we will focus on describing the role of the main onco-immunotherapies (monoclonal antibodies, cytokines, oncolytic viruses, cellular therapies, and other biological or synthetic immunomodulators) in immunogenic cell death induction and their role in modulating the innate immune system.

## 4 Dual action of immunotherapy: inducing immunogenic cell death and stimulating the innate immune response

### 4.1 Monoclonal antibodies

**Figure 1:**
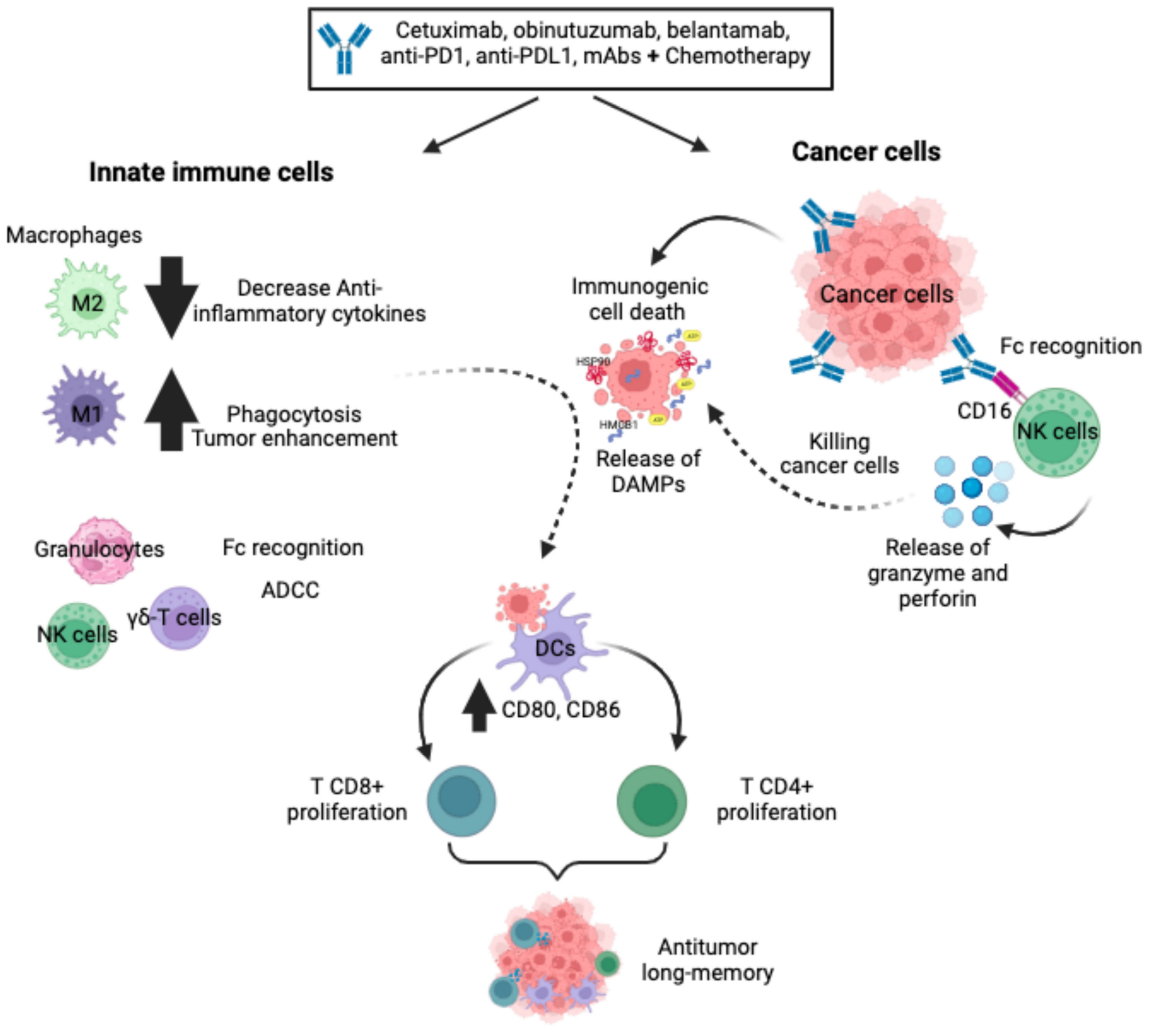
Monoclonal antibodies. The mAbs, alone or in combination with chemotherapy, improve innate cell recruitment, increase non-phagocytic tumour cell killing by neutrophils and NK cells, favour ADCC and reduce anti-inflammatory cytokines. In a variety of cancer cells, mAbs bind specifically to cancer cells, favouring Fc receptor recognition by NK cells and triggering ADCC via activating receptors. In addition, mAbs can induce ICD on cancer cells through the release and exposure of DAMPs to favour phagocytosis by DCs, triggering the stimulation of a protective T-cell (CD4+ and CD8+) memory immune response.

Monoclonal antibody (mAb)-based immunotherapies have recently emerged as one of the most important components of cancer therapy compared to surgery, radiation, and chemotherapy. Novel mAbs have been developed against neoantigens or overexpressed antigens in cancer cells that favour a variety of cell death mechanisms, including ICD (25, 26). The main clinically relevant mechanisms of action induced by mAbs on cancer cells are: antibody-dependent cellular cytotoxicity (ADCC), complement-dependent cytotoxicity (CDC), antibody-dependent cellular phagocytosis (ADCP), which involve the activation of innate cells such as natural killer (NK) cells, dendritic cells, and macrophages (27). As the aim of this review is to focus on immunotherapies that induce ICD, we will describe the principal mAbs that induce ICD and their role in innate immune responses (**Figure 1**).

Some types of monoclonal antibodies can induce ICD as monotherapy. For example, belantamab mafodotin is a humanised mAb that targets B-cell maturation antigen (BCMA) in multiple myeloma and other B-cell malignancies. The anti-BCMA is afucosylated and linked to the microtubule polymerization inhibitor, MMAF via a protease-resistant maleimidocaproyl linker. After binding to the cell surface, anti-BCMA is internalised, leading to cell-cycle arrest and apoptosis. This type of cell death triggers cell surface exposure of calreticulin (CRT), heat shock protein 70 and 90 (HSP70, HSP90), and the release of the high mobility group box protein 1 (HMGB1), adenosine triphosphate (ATP), HSP70, and HSP90, triggering activation and maturation of DCs. This leads to host innate and adaptive immune responses through tumour recruitment of cytotoxic T lymphocytes, NK cells and DCs (28). In addition, the afucosylation favours binding to FcgRIIIa receptors on the surface of immune effector cells, which promotes immune cell recruitment, activates ADCC and ADCP and generates long-term immune memory (28). Obinutuzumab (GA101), the second generation of rituximab (anti-CD20 mAb) induces ICD, which is characterised by the release of DAMPs, such as HSP90, HMGB1 and ATP, which induce DCs maturation (enhancing CD86 and CD83 expression) and subsequent T-cell proliferation (29). In addition, GA101 potentiates cellular immune responses by binding to NK cells, promoting their activation and triggering ADCC more effectively than rituximab (30). It is also able to activate γδ T-cells and potentiate killing of lymphoma cells (31), and strongly engage monocytes and M1 macrophages, leading to high levels of nitric oxide and the elimination of CD20-expressing tumour cells (32).

Although some mAbs can induce ICD as a single treatment, most reports include their combination with other immunotherapies or chemotherapies to induce ICD on multiple cancer cells. For example, cetuximab has been shown to induce increased ICD in colorectal cancer when used in combination with leucovorin calcium (folinic acid), fluorouracil, and irinotecan hydrochloride (FOLFIRI). Cetuximab induced endoplasmic reticulum (ER) stress and CRT and ERp57 expression on the cell surface, favouring phagocytosis by DCs of dying cancer cells, which triggered the stimulation of a protective T-cell (CD8+) memory immune response observed alone and in combination with FOLFIRI (33). In addition to its direct ICD induction, cetuximab is able to repolarise tumour-associated macrophages (TAMs) from M2-like to M1-like phenotypes, mainly by suppressing IL-6 expression through NFκB and STAT3 pathways (34).

Among the various antibodies used in combination, anti-PD-1 and anti-PD-L1 are the most used. For example, dinaciclib, a CDK1, -2, 5 and -9 kinase inhibitor, is a bona fide ICD inducer that, when combined with anti-PD1 mAbs, enhances DCs activation and favours antitumour response in a variety of murine syngeneic tumour models (35). Also, photodynamic therapy (PDT) enhances antitumour effects of the anti-PD-L1 mAb, inducing ICD in SCC7 cells by stimulating DCs maturation. In fact, the combination of PDT-DC vaccine and anti-PD-L1 mAb synergistically triggered an antitumour immune response and inhibited tumour progression (36). The PD-L1 mAb in combination with doxorubicin improved the immunosuppressive tumour microenvironment and promoted NK and T cell activation and proliferation. It also increased infiltrating CD8+ T cells through the secretion of CRT and HMGB1, and promoted tumour necrosis factor alpha (TNF-α) and interferon gamma (IFN-γ) production in tumour tissue in a hepatocarcinoma model (37). In addition to these effects, anti-PD-1 and anti-PD-L1 therapies increase the levels of M1-like macrophages markers and promote macrophage polarization towards the pro-inflammatory phenotype (38–40), improve NK cell anti-tumour efficacy and promote NK cell persistence and retention of their cytotoxic phenotype (41, 42). The principal antibodies that were related to ICD induction are summarized in **Table 1**.

**Table 1:** Monoclonal antibodies related to ICD induction.

mAbs	Cancer model	DAMPs	Cell death modality/characteristic	Key Result	Ref
Cetuximab alone or in combination with FOLFIRI	Panel of BRAF WT colorectal cancer cell lines	CRT and ERp57 exposure to thge cell surface	Apoptosis through ER stress	Induces phagocytosis of tumour dying cells by DCs and the induction of a protective CD8+ T cell memory immune response.	(33)
Obinutuzumab	Human lymphoma cell lines (Raji, Daudi and SU-DHL4)	Release of HSP90, HSP60, HMGB1 and ATP	Non-apoptotic programed cell death	Induces DCs maturation (enhancing CD86 and CD83 expression) and subsequent T-cell proliferation.	(29)
Belantamab	Multiple Myeloma (NCI-H929 cells)	Exposure and release of CRT, HSP70, HSP90, HMGB1 and release of ATP	Apoptosis	Induces cell-cycle arrest and apoptosis and promotes the recruitment of immune cells leading to ADCC and ADCP.	(28)
The PD-L1 mAb in combination with doxorubicin	Mouse hepatocarcinoma cell lines Hepa1-6 and H22	Release of CRT and HMGB1, TNF-α and IFN-γ production in tumour tissues	Apoptosis through cell cycle arrest	Improves tumour immunosuppressive microenvironment and promotes the activation and proliferation of NK and T cells. Also, increased CD8+ T cells infiltration	(37)
Anti-PD1 mAb combined with Dinaciclib	Mouse colon adenocarcinoma in MC38 cell line	Release of CRT, HMGB1 and ATP	Not described	Enhances DCs activation and antitumour activity in several murine syngeneic tumour models.	(35)
Anti-PD-L1 mAb combined with Photodynamic therapy	Squamos cell carcinoma SCC7 cells	Release of CRT, HMGB1 and ATP	Not described	Stimulates DCs maturation, induces antitumour immunity, and suppresses tumour progression.	(36)

Monoclonal antibodies can also activate cells of the innate immune system, such as NK cells, and trigger ADCC via NK cell-activating receptors, such as CD16. Even polymorphonuclear granulocytes such as eosinophils, neutrophils, macrophages, and monocytes can engage in Fc-mediated effector functions against antibody-opsonized tumour cells through multiple mechanisms (43, 44).

Several antibody therapeutics approved by the Food and Drug Administration (FDA) for various haematological and solid cancers have been reported (45), and have shown to activate innate immune cells. Among these, Rituximab was the first FDA approved mAb against B-cell lymphomas; its therapeutic activity in combination with chemotherapy improves innate cell recruitment (46). Human anti-CD47 antibodies enhance nonphagocytic tumour cell killing by neutrophils and NK cells in an acute myeloid leukaemia (47). Enavatuzumab is a humanized IgG1 antibody that exerts potent ADCC on TweakR positive tumour cells by monocytes and NK cells *in vitro* (48). Monalizumab, a humanized anti-NKG2A antibody, increased NK cell activity against cancer cells and established CD8+ T cell function in BALB/c mice bearing B cell lymphoma A20 cells *in vivo* (49).

The potential of mAbs to induce innate antitumour immune responses suggests that it may only be a matter of time before we fully exploit their capabilities to orchestrate a collaborative effort between innate and adaptive immune responses against cancer, ultimately generating long-term antitumour memory (**Figure 1**).

### 4.2 Cytokines

Cytokines are molecules that play a central role in cellular autocrine or paracrine signals that are released or produced in response to various stimuli, leading to differentiation, proliferation, activation, cell death and other effects. Cytokines also regulate innate and acquired immune responses such as pro- and anti-tumour effects. Thus, cytokine-based immunotherapies are a promising therapeutic approach that can be used to promote, enhance, maintain or regulate the establishment of an anti-tumour immune system (50).

In general, it has been reported that IL-2, IL-7, IL-12, IL-15, IL-18, and IL-21 induce the expansion and enhance the cytotoxicity of NK, NKT and T lymphocytes, whereas granulocyte macrophage colony-stimulating factor (GM-CSF) and granulocyte-colony stimulating factor (G-CSF) promote the expansion and activation of DCs, and most of them have recently been evaluated in various clinical trials (50, 51). However, most of the cytotoxic or antitumour evaluations of cytokines are in combination with other agents and the direct cytotoxic effect of cytokines in tumour cells is poorly evaluated.

In this sense, the combination of TNF-α and secondary mitochondria-derived activator of caspases (SMAC) mimetics has been reported to induce immunogenic cell death in fibrosarcoma, melanoma, liposarcoma, synovial sarcoma and patient-derived cutaneous squamous cell carcinoma. *In vivo*, the combination of TNF-α, SMAC mimetics and melphalan induced tumour shrinkage, promoted the activation of CD8+ T cells as well as NK cells and prolonged survival in a rat model of liposarcoma (52).

On the other hand, a cytokine-triggered inflammatory cell death pathway involving crosstalk between the machinery of pyroptosis, apoptosis and necroptosis cell death, termed PANoptosis, has recently been reported. Subbarao et al. showed that a cocktail of pro-inflammatory cytokines including TNF-α, IFN-γ, IL-1α, IL-1β, IL-18, IL-6, IL-8, and IL-15 induced cell death in NCI-60 colon cancer cells, while individual treatments or a cocktail lacking TNF-α and IFN-γ did not induce cell death, suggesting a synergistic effect of this cytokines signalling to induce cell death. The unique combination of TNF-α and IFN-γ induced PANoptosis in different cancer cell types such as colon, melanoma, lung cancer and leukemic cell lines, highlighting the robust cell death induction by TNF-α and IFN-γ in a wide range of cancer cells. Interestingly, the intratumorally administration of a combination of TNF-α and IFN-γ suppresses the tumour growth in a human colon cancer model (53), while independent treatment does not induce these effects. However, although pyroptosis and necroptosis are associated with immunogenicity, immunogenic cell death has not been assessed, but these reports shed light on the possibility that these combinations could lead to ICD.

### 4.3 Oncolytic viruses

**Figure 2:**
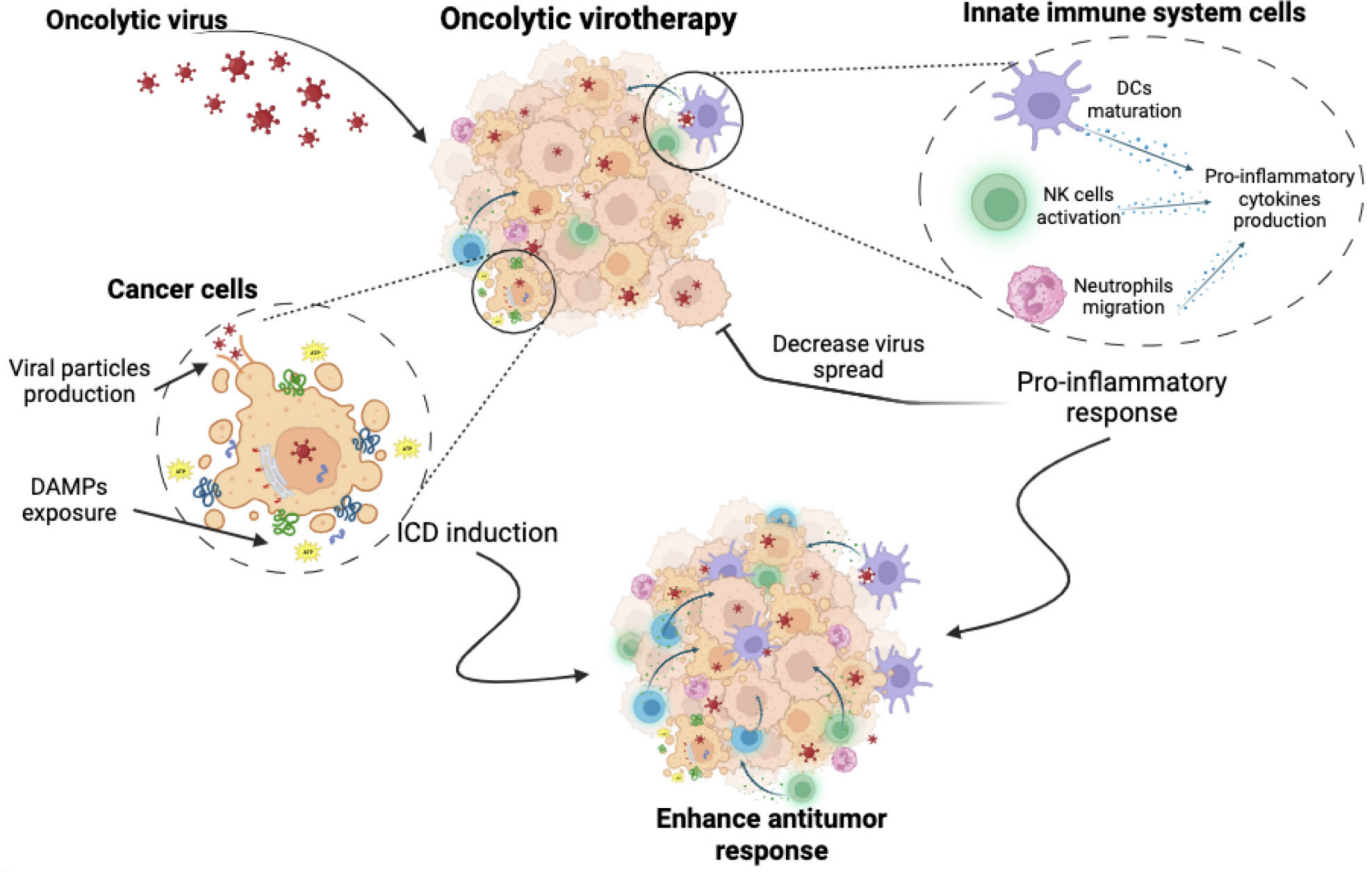
Oncolytic virotherapy. Oncolytic virotherapy has a dual antitumour effect. It induces a direct cytotoxic effect, causing cancer cell death through mechanisms involving the exposure and release of DAMPs, the production of viral particles and finally the induction of immunogenic cell death. At the same time, oncolytic virus triggers the activation of innate immune cells, leading to the stimulation of pro-inflammatory responses. While this may enhance the anti-tumour immune attack, it could also inhibit the virus spread, which could pose a challenge to treatment efficacy.

Oncolytic viruses (OVs) are a novel immunotherapy strategy using competent or genetically modified oncolytic viruses that selectively infect, replicate, and induce cell death in tumour cells. OVs have a unique mechanism of action, combining direct tumour cell death, tumour-specific immune response, and antiviral immune system activation. OVs induce oncolysis through the production of viral particles that spread to surrounding tumour cells and promote immune system activation through the release of PAMPs, DAMPs, viral particles and neoantigens (54, 55) (**Figure 2**). OVs have been reported as ICD inducers and, depending on the type of virus (adenovirus, herpes simplex, semliki forest virus, vaccinia virus, reovirus, among others), they can induce cell death by different mechanisms. These cell death mechanisms include apoptosis, necroptosis, pyroptosis and autophagic cell death, but in general they all induce the exposure and release of DAMPs and PAMPs (54, 56).

Currently, a diverse array of OVs has undergone extensive evaluation of their ability to induce ICD across a wide spectrum of tumour models. Notable examples include adenovirus OBP-702 in pancreatic cancer (57), adenovirus dl922-947 in mesothelioma (58), talimogene laherparepvec, and measles virus in melanoma (59), adenovirus serotype 5, semliki forest virus, and vaccinia virus in osteosarcoma (60), reovirus type 3 Dearing strain in lymphoma and prostate cancer (61), adenoviruses, Ad884 and Ad881 in colon cancer (62), oncolytic Newcastle disease virus (NDV) in human lung cancer (63), while herpes virus H-1PV, RH2, and VC2 in pancreas, squamous carcinoma, and melanoma cancer cells (64–66) (**Table 2**).

**Table 2:** Oncolytic virus eliciting immunogenic cell death.

Virus	Cancer model	DAMPs	Cell death modality/characteristic	Antitumour effect and immune system involvement	Ref
Adenovirus OBP-702	Human Pancreatic ductal adenocarcinoma cells (PDAC) with different p53 status (Capan-2, PK-59, PK-45H, Capan-1, MIA PaCa-2, BxPC-3) and murine PDAC cells (PAN02)	ATP and HMGB1	Increased the expression of p53, cleaved PARP, decreased the expression of p62	Tumour infiltration of CD8+ T cells and CD11c dendritic cells	(57)
dl922-947	Malignant mesothelioma cell lines MSTO-211H and NCI-H28	ATP, HMGB1 and Calreticulin	Necroptosis	Inhibits tumour growth and reduces the tumour micro-vessel density (TMD)	(58)
Talimogene laherparepvec (T-VEC)	Human melanoma cell line SK-MEL-28	ATP, HMGB1 and Calreticulin	Cleaved caspase-3 and PARP	Increased of CD3+ and CD8+ T cells, and induces a systemic pro-inflammatory gene signature	(67)
Wild-type human Adenovirus serotype 5 (Ad)	Human bone osteosarcoma cell line HOS and human lung carcinoma cell line A549	ATP, HMGB1, Calreticulin and HSP90	RIP3 and MLKL activation, Inflammasome assembly and mature IL-1β, autophagosome formation	Increased DCs phagocytosis and maturation, activation of antigen specific T cells	(60)
Semliki Forest virus (SFV) strain4	Human bone osteosarcoma cell line HOS and human lung carcinoma cell line A549	ATP, HMGB1, Calreticulin and HSP90	Cleaved caspase-3/7 and caspase-8, autophagosome formation	Increased DCs phagocytosis and maturation, activation of antigen specific T cells	(60)
Vaccinia virus (VV) Western Reserve stain	Human bone osteosarcoma cell line HOS and human lung carcinoma cell line A549	ATP, HMGB1, Calreticulin and HSP90	Activation of MLKL	Increased DCs phagocytosis and maturation	(60)
Measles virus	Human melanoma cell lines Mel888, Mel624, SkMel28 and MeWo	Not determined	Not determined	Increased the activation marker CD69 and degranulation marker CD107a in NK cells. Promoted DCs maturation and T CD8+ cells priming.	(59)
Reovirus type 3 Dearing strain	B cell lymphoma (Daudi) and bladder (EJ) tumour cell lines Prostate cancer-derived cell lines PC-3, DU145 (human), and TRAMP-C2 (Murine)	Not determined ATP, HMGB1 and Calreticulin	Not determined Not determined	Promoted DCs maturation and proliferation of T cells and enhance NK cells anti-tumour-cytotoxicity Promoted the survival of TRAMP-C2-bearing C57BL/6 mice, and increased the CD4+ expressing IFN-γ cells and promotes antitumour memory	(61, 68) (61, 68)
Reovirus type 3 Dearing strain-mutant jin-3	Human prostate cancer cell lines PC-3M-Pro4luc2, DU145, and 22Rv1	Not determined	Cleaved caspase-3	Decreased tumour burden and tumor volume. Increased the expression of the inflammatory cytokines CXCL10, TNF-α, and IL-1β.	(69)
HSV-P10	Murine breast cancer cell lines DB7, Met-1, and MVT-1 and human MDA-MB-231, SK-BR-3, MCF-7, and MDA-MB-468	Not determined	Not determined	Increased mice survival and induced antitumour immune memory of mice bearing breast cancer brain metastases. Induced intratumoral infiltration of macrophage, DCs, NK and CD8+ cells.	(70)

These viruses have demonstrated their capacity to trigger the release of critical DAMPs, such as ATP, HMGB1, calreticulin, HSP70, and HSP90 from dying cancer cells. This release enhance the phagocytosis and maturation of DCs, facilitating the infiltration of cytotoxic T cells, and bolstering the NK cell’s antitumor activity, particularly notable in the case of the measles virus, reovirus type 3 Dearing strain, and HSV-P10 (59, 61, 70). This immunogenic response leads to the secretion of pro-inflammatory cytokines such as IFN-γ, TNF-α. **Table 2** offers a comprehensive compilation of various research studies delving into the utilization of oncolytic viruses as potent inducers of immunogenic cell death.

In addition to their primary role in killing cancer cells, OVs also serve as potent activators of the innate immune system (71). This is because OVs are pathogens specifically designed to infect and destroy cancer cells. When OVs infect tumour cells, they elicit an inflammatory response, leading to the localized production of cytokines and chemokines that promote the stimulation of the innate immune response trough different mechanisms. These mechanisms include the activation and recruitment of neutrophils (72), macrophages, and NK cells (71). Furthermore, OVs can initiate the activation of PRR in innate cells, triggering the activating receptors such as toll like receptors (TLRs).

Thus ICD-induction by OVs plus the antiviral immune response activated by OVs, promote an immune-stimulatory environment, leading to the uptake of TAAs and neo-antigens by PRR stimulated antigen presenting cells (APCs). Altogether, these events result in a dual immune system activation, on the one hand the generation of immune responses against virally infected cancer cells, and in the other hand immune responses against TAAs and neo-antigens of un-infected cancer cells. Thus, the ‘indirect’ effects of the antiviral immune response within the tumour site, including the release of pro-inflammatory cytokines and the cytotoxicity of infected tumour cells, can reverse the immunosuppressive TME. This in turn may enhance ICD-related properties, including stimulation of innate and adaptive immune cells, release of pro-inflammatory cytokines, and recruitment of immune cells into tumours (63, 73). The anti-viral immunity triggered against viral antigens from the resultant infection is also a key player during OV-based therapies, as tumour cell infection promotes the antiviral immune response, which can be seen as a negative response triggered against OVs, but it helps to settle an inflammatory site that turns “cold” tumours “warm” (74).

However if unbalanced, this immune response could induce premature clearance of OVs and compromise their antitumour efficacy (75), as it has been observed in herpes simplex virus (oHSV) therapy, where activated NK cells reduce the anti-tumour efficacy of HSV in glioblastoma cells (76). In this regard, the combination of transforming growth factor beta (TGFβ) and oHSV therapy inhibits NK cell recruitment and function, resulting in enhanced viral replication in glioblastoma mouse models (77). In addition, the downregulation of the NK cell-activating ligand CD155 inhibited NK cell recruitment *in vitro*, enhancing viral replication in a rat model of hepatocellular carcinoma (78). IFN-γ and TNF-α secretion also induce virotherapy resistance in different animal models (79, 80), being IFN-γ signalling modulators/inhibitors a strategy used to improve the success of OV treatment (81).

In summary, oncolytic viruses exert a wide range of direct and indirect antitumour effects, including tumour oncolysis, induction of tumour-specific immune responses and activation of the antiviral immune system. These effects culminate in the effective generation of neoantigens and the release of DAMPs and PAMPs, which in turn promote the recruitment of neutrophils, granulocytes, NK cells and APCs to the site of viral replication (56, 72, 81). This in turn enhances the activation of the T- and B-cell-mediated adaptive immune response (56). Finally, these combined effects can potentiate the anti-tumour immune response, which is further enhanced by the induction of ICD, ultimately leading to the establishment of anti-tumour memory.

### 4.4 Cellular immunotherapies

Cellular immunotherapy, or adoptive cell therapy, is a form of treatment that involves the infusion of live cells into a patient’s body to eliminate cancer. Some of these approaches involve the direct isolation and subsequent expansion of immune cells (T cell-based, NK cell-based, macrophage cell-based, and DC-based), while others use genetic engineering techniques, such as gene therapy, to enhance their cancer-fighting potential (such as chimeric antigen receptor (CAR)-based immunotherapies). CAR-based immunotherapies involve the genetic modification of the cells to confer tumour specificity and include CAR T cells, which comprise the innate T cell subsets such as the γδ T cells (γδCAR T cells) and natural killer T (CAR NKT), CAR NK, and CAR macrophages, which together enhance the potential of cellular immunotherapies (82, 83). In this section, we will focus on the role of cellular immunotherapies in ICD and the activation of innate immune cells.

#### 4.4.1 CAR-based immunotherapies

##### 4.4.1.1 CAR T-cell therapies

CAR T-cell therapy is a highly promising and rapidly advancing treatment approach primarily for haematologic malignancies (84, 85). CAR T cells eliminate cancer cells by binding to target cell surface antigens without the need for MHC restriction. Many articles have reviewed broadly their use, limitations and potential strategies (86–89), while in this review we will principally approach their possibility as ICD inductors, and CAR innate T cell subsets.

Although T cells predominate in CAR-based immunotherapies, innate T cell subsets can also be used for CAR redirection, such as γδCAR T cells and CAR NKT cells. It has been proposed that CARs in innate cells may be preferable than CAR T cells, because of a reduced cytokine release storms (CRS) and graft versus host disease (GvHD). CAR NKT cells simultaneously express the invariant TCR in addition to the CAR, thereby preserving their responsiveness to glycolipid antigens (90). CAR NKT cells in addition to presenting effects on liquid tumours like B-cell lymphoma and multiple myeloma, through CD19, CD38/BMCA, CEA, or HER2 targeting (91, 92), they can also target solid tumours like GD2 in neuroblastoma (93), CSPG4 in melanoma (94) due to their unique capabilities, like high infiltration into TME (91). On the other hand, γδCAR T cells can respond to CD19-positive and -negative tumour cells, suggesting that CD19-directed γδCAR T cells can target leukemic cells even after antigen loss (95). In addition to CD19 targeting, γδCAR T cells have also shown interesting results when targeting glypican-3 in hepatocellular carcinoma (96). In addition, γδCAR T cells produce less IFN-γ and other inflammatory cytokines when compared to conventional αβ CAR T-cells, which may result in a lower risk of CRS (97).

CAR T cells mediate their anti-tumour effects using similar mechanisms as native T cells, such as granular exocytosis and expression of death ligands (98). These mechanisms lead, in native T cells, to different cell death modalities that include apoptosis, necroptosis, pyroptosis, and ferroptosis (99), and can lead to immunogenic cell death in target cancer cells (100, 101). T lymphocytes promote calreticulin exposure, HMGB1 and IL-1β release, leading to DC uptake and cross presentation, providing a mechanism for amplification and self-perpetuation of the immune response against cancer neoantigens (100, 101). They also release cytokines that sensitise the tumour stroma and promote inflammatory signalling, such as IFN-γ which has also the ability to sensitize to cell death and directly trigger cell death alone or combined with other inflammatory molecules, such as TNF-α (99). Although there are no reports specifically describing whether CAR T, γδCAR T or CAR NKT therapies can induce ICD on cancer cells, it is likely that this could occur, as they share common mechanisms of cytotoxicity.

##### 4.4.1.2 CAR NK Cells

Natural killer cells are a vital component of the innate immune system, serving as the frontline defence against infected, transformed, and stressed cells. NK cell activation is initiated by stimulation of an activating receptor, often NK46 encoded by NCR1 (102, 103). Upon activation, NK cells release cytotoxic granules that contain perforin and granzymes to directly lyse cells (104, 105), or regulate the adaptive immune responses by releasing chemokines and cytokines such as IFN-γ and TNF-α. Importantly, NK cells are critical for tumour immunosurveillance, as increased cancer susceptibility and metastasis have been reported in mouse models and clinical trials with low NK activity (106, 107). Due to their inherent ability to target and destroy cancer cells, NK cell-based immunotherapies have been investigated for cancer treatment for decades, including therapies such as CAR NK cell therapy.

A wide range of tumour antigens have been targeted by CAR NK cells in pre-clinical studies for haematological malignancies and solid tumours (108). As for CAR T therapies CD19 is the most common target in CAR NK cells in both preclinical and clinical studies. Also, molecules such as CD20 and Flt3, have been developed as specific targets of CAR-NK against B-cell tumours (109), while CD38, CD138, B-cell maturation antigen, and signalling lymphocytic activation molecule family member 7 have been developed against acute myeloid leukaemia, and CD3, CD5 and CD7 for the cases of T-cell malignancies (110–113). Interestingly, in another therapeutic approach, CAR NK cells seek to eliminate myeloid-derived suppressor cells (MDSCs) (114) and M2TAMs (115) to reverse the immunosuppressive tumour microenvironment.

While detailed analyses of cell death induced by CAR NK cells have not been conducted, it is plausible that they share similar mechanisms with conventional NK cells, which execute cellular cytotoxicity through granule exocytosis and death ligands. NK cells possess the versatile ability to activate diverse cell death pathways, including apoptosis, necroptosis, and pyroptosis, and they are also capable of mediating immunogenic cell death by enhancing dendritic cell uptake of dying cells and facilitating antigen cross-presentation, ultimately leading to the development of immunogenic memory (101, 116). Similar to CAR T cells, there are no reports describing whether CAR NK therapies can induce ICD on cancer cells, but it is likely that this mechanism could also be applicable.

##### 4.4.1.3 CAR macrophages

CAR macrophages (CAR M) are widely recognised as a potential treatment for solid tumours due to their prominent functions in immune regulation and their ability to infiltrate solid tumours. They are currently under clinical investigation as they retain phagocytic and M1 functions while migrating to both primary and metastatic tumours (117). Tumour antigen-specific CARs show significantly enhanced cytotoxicity against tumour antigen-expressing cells and have the potential to remodel the tumour microenvironment (118). CAR constructs used in CAR M cells principally include CD19 in non-solid tumours (119), HER 2 in breast (120) and ovarian cancer cells (121), and mesothelin in ovarian cancer cells (121).

The molecular mechanisms underlying the anti-tumour activity of macrophages are not fully understood. It has been established that macrophages have the ability to eliminate cancer cells through multiple mechanisms, including (1) indirect killing by recruiting cancer cell-killing immune cells such as innate (NK) and adaptive (T) cells (2), cytolysis through antibody (Ab)-dependent cellular cytotoxicity, and (3) direct cancer cell killing by releasing oxygen radicals such as nitric oxide, reactive oxygen species, IL-1β, and TNF-α (122). Although nitic oxide (NO), reactive oxygen species (ROS), IL-1β and TNF-α mediated cell death has been studied in cancer cells and may be associated with ICD induction, this cell death mechanism has not yet been elucidated in macrophage mediated cell death.

### 4.4.2 Dendritic cells

Dendritic cells are the major APCs that form the link between the innate and adaptive immune systems. These cells efficiently process and present antigens via histocompatibility complex I and II molecules to both innate and adaptive immune cells, thereby triggering the activation of both cellular and humoral immune responses (123).

In addition, DCs play a central role in the activation of the antitumour response during immunogenic cell death. Following the induction of cancer DAMPs exposure or release by various treatments (anthracyclines, oncolytic viruses, anticancer peptides, among others), DCs can be stimulated by different pathways (**Table 3**).

**Table 3:** The impact of DAMPs in Dendritic cells.

DAMPs	Cell receptor	Effect in DCs	Ref
ATP	P2X7, P2Y2	Intracellular Ca2+ increase, actin rearrangement, chemotaxis, migration, activation of NLRP3 inflammasome and release of IL-1β	(124–126)
Calreticulin	CD91	Promotes the phagocytic activity and the release of pro-inflammatory cytokines	(125–128)
HSP70 and HSP90	CD91, TLR4	Promotes the antigen cross presentation, and enhances the processing and presentation of antigens	(129, 130)
HMGB1	TLR2, TLR4 and RAGE	Stimulates the generation of pro-inflammatory cytokines while simultaneously aiding in effective antigen presentation and promotes cross presentation	(131–133)

Due to the immunostimulatory effects of DCs, and their crucial role in the presentation of TAAs, DCs are an excellent means of enhancing the body’s natural anti-tumour responses. Therefore, DC-based immunotherapy focuses on harnessing the potential of DCs to effectively present tumour antigens and induce targeted anti-tumour immune responses.

In DC-based immunotherapy, the source of the DCs (peripheral blood monocytes, haematopoietic precursors, peripheral blood enriched DCs, etc.) and the stimulation with the antigen are crucial steps for the efficacy of the therapy. In addition, different sources of TAAs such as: whole tumour lysates, synthetic peptides, purified tumour antigens, genetically engineered DCs, among others, and different antigen-loading methods are used for stimulation (134). Interestingly, a very important source of TAAs for DC vaccines are cancer cells killed by ICD inducers or strategies. In particular, whole tumour vaccines are crucial for the stimulation of long-term anti-tumour immune responses by DC vaccines, as they serve as a potential reservoir of tumour antigens and could lead to enhanced anti-tumour T-cell responses (135). DCs vaccines loaded with doxorubicin-treated tumour cells are effective in a prophylactic application by reducing tumour development in neuroblastoma (NXS2) and melanoma (B16F10) cell-bearing mice in a prophylactic and therapeutic setting, respectively (136, 137). Also, a DCs vaccine loaded with shikonin (an ICD inductor) treated melanoma cells significantly promoted tumour reduction and improved survival of mice in a therapeutic application (137). Additionally, DCs stimulated with shikonin-treated breast cancer (4T1) cells suppressed metastasis and increased survival of breast cancer cells in an orthotopic tumour resection model (138).

On the other hand, although ICD was initially conceived as a form of chemotherapy-induced tumour cell death, physical anticancer approaches (radiotherapy, photodynamic therapy, among others) have demonstrated the capacity to generate an immune response that can be exploited in DC-based vaccine strategies (125). In this sense, it has been reported that DCs vaccines stimulated with killed squamous cell carcinoma cells by PDT promote tumour reduction and increase the survival of mice showing a better response that the application of tumour cell lysate (139), indicating that the use of DCs enhance the antitumour response. In addition, PDT-based DCs vaccine inhibits the growth of mesothelioma tumours and increases the survival of mice (140).

Furthermore, immunotherapy with DCs is currently being used in combination with ICD inducers. Mice treated with DCs and doxorubicin show an increase in CD8+ T lymphocytes within metastatic tumours and inhibition of metastatic growth (141). Similarly, an increase in serum IL-2, IL-12 and IFN-γ, as well as the proportion of IFN-γ+ CD8+ T cells, was observed in a randomized trial of oesophageal cancer patients treated with DCs vaccine and radiotherapy (142).

Finally, despite the diverse reports of DCs vaccine effects, most of the DCs activities in immunotherapy focus on the activation of the adaptive immune system. However, there are reports on the effect of DCs in different cells of the innate immune system. DCs activate and potentiate the cytotoxic activity of NK cells via IL-12, IL15 and IFN-γ (143). In addition, DCs can activate NKT cells through the expression of invariant CD1 molecules and the presentation of glycolipids (144). Thus, DCs play a critical role in the activation of both innate and adaptive immune responses. However, the activation of innate immune cells by DCs is poorly understood and further evaluation in the context of ICD induction is needed to expand the knowledge of the effect of DCs and to propose more efficient combinatorial treatments.

### 4.5 Other immunomodulators

#### 4.5.1 Biological immunomodulators

Biological immunomodulators, also called biological therapies are a subset of immunotherapies obtained from biological entities, such as bacillus Calmette-Guérin (BCG), an attenuated *Mycobacterium bovis* derivative, and dialyzable leukocyte extracts, obtained from immune cells, among others. They are used in several diseases such as autoimmune diseases, viral and bacterial infections and recently in cancer (145, 146).

Bacille-Calmette-Guerin (BCG) is a live attenuated tuberculosis vaccine that is widely used in neonates to induce long-term immunity against pathogens such as *Mycobacterium tuberculosis*, *Candida albicans* and *Staphylococcus aureus* (147). Few reports have described its involvement in the cancer immune response. In this sense, it has been reported that it induces caspase-independent cell death with the release of HMGB1 into the extracellular space in a dose-dependent manner in urothelial carcinoma (UC) T24 and 253J cell lines. The authors also found urinary levels of HMGB1 in patients diagnosticated with UC at 24 hours after BCG therapy (148). In other hand, BCG vaccination triggers innate immune training in several types of immune cells, including monocytes, neutrophils, NK cells and dendritic cells. This training occurs through the interaction of various PRRs with PAMPs present in the bacterial cell wall (149). Immediately, innate cells respond by secreting pro-inflammatory cytokines, including IL-6 IL-1β, TNF-α, monocyte chemoattractant protein- 1 (MCP-1), and IL-8 (150). Consequently, cellular infiltration of T cells (CD3+), monocytes (CD14+), but predominantly CD15+ neutrophils occur at the vaccination site (151). *In vitro* studies show that human blood neutrophils obtained from BCG vaccination sites cooperate with dendritic cells to enhance antigen-specific T-cell responses (152)Indeed, BCG enhances innate immunity in the context of pathogen protection, however the implication of BCG vaccination and anti-tumour immunity is not yet described (148).

Other types of biological immunomodulators are animal extracts derived from the immune system. This group includes substances produced by immune system cells, also known as dialysable leukocyte extracts (DLE). DLE are a diverse mixture of low-molecular weight compounds derived from blood or lymphoid tissue with immunomodulatory properties (153). Several reports have shown that DLE derived from human blood or lymphoid tissue from different animals (crocodile, porcine or bovine) can regulate numerous molecular targets, thereby facilitating immunomodulatory effects in conditions such as autoimmune diseases, immunodeficiencies, asthma, bacterial infections and certain types of cancer (153–156). The bioactive peptides contained in DLE, irrespective of their source species, have displayed analogous effects on both mouse and human leukocytes, involving the activation of comparable signalling pathways associated with their immunomodulatory properties (155, 157). Recently, DLE have been shown to induce cytotoxicity in several cancer cell lines (156, 158–161).

**Figure 3:**
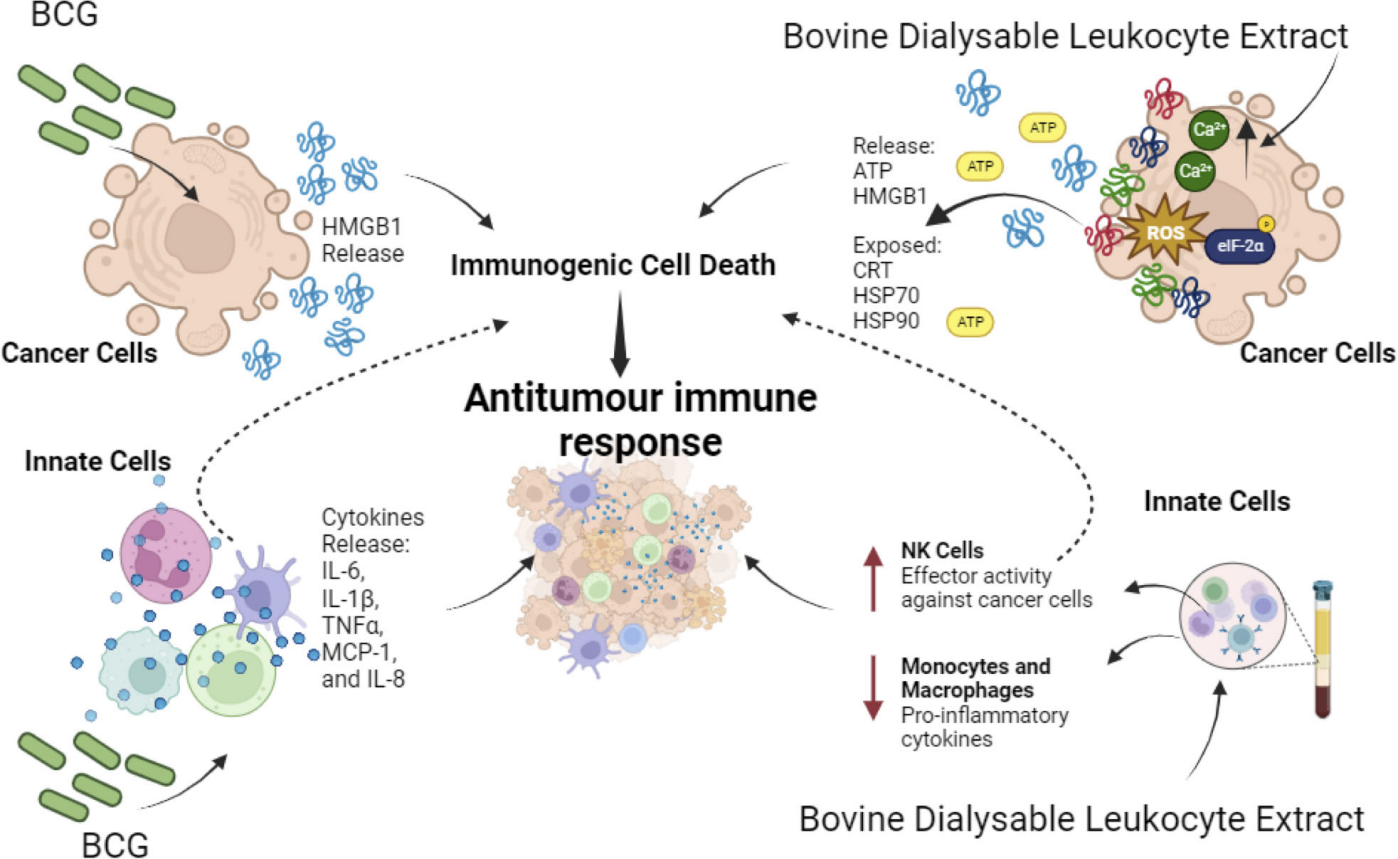
Effect of Biological Immunomodulators on cancer cells and innate immune cells. BCG induces HMGB1 release in cancer cells that promotes ICD. Also, on innate immune cells it induces pro-inflammatory cytokines’ release IL-6, IL-1β, TNF-α, MCP-1, and IL-8. On the other hand, bovine dialysable leukocyte extracts induce cytotoxicity on a variety of cancer cells, in most cases through immunogenic cell death induction, thereby enhancing antitumour immune responses. In parallel it enhances effector activity on NK cells against cancer cells while decreasing proinflammatory cytokines on macrophages and monocytes during lipopolysaccharide (LPS) stimulation.

Immunepotent CRP (I-CRP) is a DLE obtained from bovine spleen (bDLE), which has a wide range of applications in humans. Several studies have shown that it can modulate human and murine immune cells, while inducing cytotoxicity against human and murine tumour cell lines. In particular, its cytotoxic effect has been demonstrated in lung cancer (161), breast cancer (156, 159, 160), murine lymphoma (146), cervical cancer (160, 162) and leukemic cell lines (163). Currently, in a murine melanoma model, I-CRP has been shown to increase the release of DAMPs and the immunogenicity in combination with oxaliplatin (164). It also induces ICD in a murine breast cancer model, involving the DCs maturation in lymph nodes and the increase of CD8+ T cells in lymph nodes, peripheral blood and tumour site, favouring long-term memory (156). On the other hand, in human PBMC, ICRP increased the CD56Dim CD16- subset and modulated NKp30, NKp44, NKp46, NKG2D, NKG2C and KIR receptors, whereas there were no significant differences in CD160, CD85j and CD226 in human NK cells. These alterations revealed increased antitumour cytotoxic activity due to changes in the receptor repertoire of NK cells (155) (**Figure 3**).

Another type of DLE, derived from human blood cells (Transferon), suppressed tumour growth and promoted the differentiation of haematopoietic stem/progenitor cells into CD56+CD16+CD11c+ NK-like cells capable of eliminating tumour cells and stimulating the proliferation of γδ T lymphocytes (165). Besides, it decreased metastatic dissemination of intracardiac prostate epithelial cells and prevented tumour establishment of subcutaneous isotransplants. This effect has been associated with high levels of IL-12 and CXCL1, diminution of VEGF levels and changes in tumour infiltration of mononuclear cells and neutrophils (158). Also, Immodin, another human DLE, in combination with manumycin A suppressed tumour growth and prolonged survival in mammary tumour-bearing mice. This combination increased the infiltration of neutrophils and eosinophils into the TME, while independent treatments increased the phagocytic activity of monocytes and neutrophils (166). However, the immunogenicity of cell death has not been evaluated. In **Figure 3** we can depict the effect of biological immunomodulators in cancer cells and innate immune cells.

#### 4.5.2 Synthetic immunomodulators

Synthetic immunomodulators are chemical agents that can be derived from diverse sources and can modulate biological responses by interacting with specific cellular targets (167, 168). Synthetic immunomodulators, which may encompass peptides and small molecules, have been used for decades (167–170). They are now being employed in cancer immunotherapy, with a focus on targeting specific surface molecules on cancer cells. These compounds can directly influence signalling pathways and modulate immune cells to selectively target specific types of cancer cells (168, 170). Additionally, some of them show a cytotoxic effect and their role as ICD inducers has recently been explored.

One of these molecules is imiquimod (IMQ), the first member of the immune response modifier family to be approved by the FDA in 1997, for the treatment of external genital and perianal warts (171, 172). IMQ activates toll like receptor 7 (TLR7), which is overexpressed in different types of cancer (173), it also has potent antiviral and antitumour effects as shown in preclinical and clinical studies. Specifically, in human peripheral blood mononuclear cells (PBMC), IMQ has been shown to increase cytokine production including IFN-γ, TNF-α, IL-1, IL-6 and IL-12 by macrophages and monocytes. IMQ also stimulates NK cell activity against skin-infected cells and the activation of macrophages to produce nitric oxide (171, 174). Also, in acute and chronic infectious diseases it promotes anti-inflammatory molecules such as IL-10, and indoleamine 2,3-dioxygenase (IDO) (175–177).

**Figure 4:**
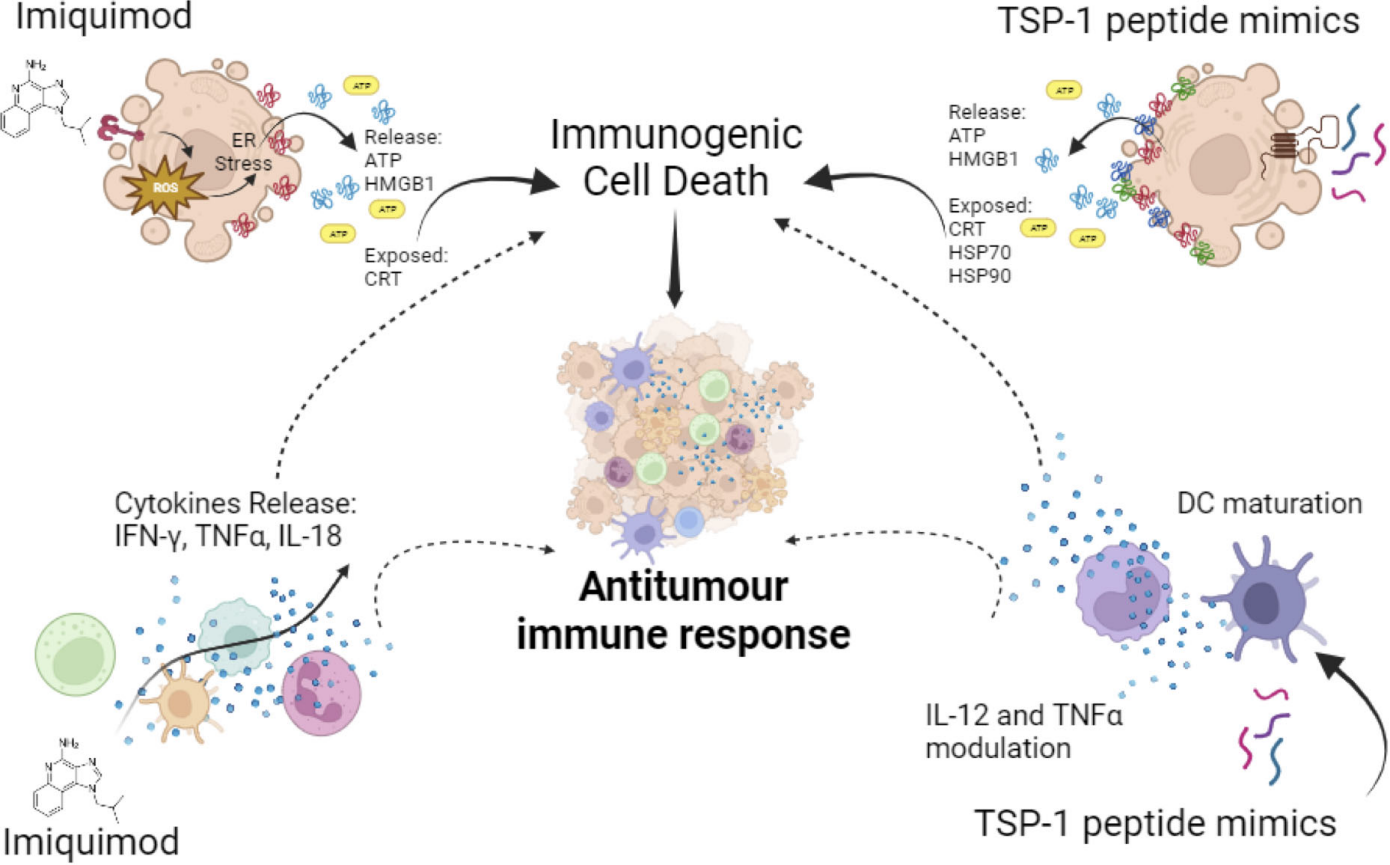
Synthetic immunomodulators in cancer cells and innate immune cells. Imiquimod induces ATP and HMGB1 release and CRT exposure, leading to ICD in cancer cells, also on innate immune cells it induces pro-inflammatory cytokines’ release favouring antitumour immune responses. TSP-1 peptide mimics induce cytotoxicity in a variety of cancer cells leading to ICD, through CRT exposure and ATP, HMGB1, HSP70 and HSP90 release, also in immune cells it induces the modulation of IL-12 and TNF-α and DC maturation. All these effects promote antitumour immune responses.

In the context of cancer cells, it can directly induce tumour autophagic cell death in melanoma (178), breast cancer (179) and colorectal cancer (180). It has also been shown that IMQ induces ICD by promoting ROS production, which triggers ER stress followed by surface-exposed CRT, ATP secretion and HMGB1 release (**Figure 4**), in BCC/KMC-1, AGS, HeLa and B16F10 cancer cells (181). Vaccination with IMQ-killed cancer cells also increased T lymphocyte proliferation, cytotoxic killing and immune cell infiltration into the tumour lesion in an *in vivo* melanoma model (181). In transgenic mice IMQ promoted breast cancer tumour regression, which progressed at the end of treatment due to CD4+ cells augmentation that enhanced IL-10 levels (182). On the other hand, IMQ could also improve the antitumour immune response by MAA peptide-pulsed DC immunotherapy (183). These effects are due to the stimulation of TLR7 in tumour cells and seem to depend on the type of cancer, the level of TLR7 expression, the downstream function of TLR7 signalling, or chemotaxis of suppressive cells into the tumour (173). Thus, although IMQ has promising cell death inducing and immunomodulatory effects, caution should be taken about these contrary effects reported.

Other synthetic molecules such as thalidomide, lenalidomide and pomalidomide have demonstrated cytotoxicity in a variety of cancer subsets, however their activity as ICD inductors have not been described (184, 185). Recently, clinical trials have reported improved antitumour responses in multiple myeloma when used alone or in combination with other immunomodulatory agents (186–188). Thalidomide, in particular, was originally synthesised in the late 1950s as a non-addictive, non-barbiturate sedative (189). It is clinically useful in a number of cancers because of its antitumour activity, which is related to the secretion of various cytokines, including IL-2 and IFN-γ as well as inducing T-cell costimulatory and antiangiogenic activities (189, 190). These reports suggest that these molecules can provide satisfactory stimulation of innate immune cells and contribute to cancer elimination through ICD induction.

##### 4.5.2.1 Peptide-based immunotherapies

Peptides are short-chain molecules, typically consisting of less than 50 amino acids. They have applications in the treatment of various conditions such as allergies, infections, tumours, and other diseases. Some peptides can induce cell death in bacteria, fungi, and tumour cells. In particular, peptides are gaining prominence in the field of immunotherapy due to their significant impact on the immune system (191). Therapeutic peptides have found application in immunotherapy, serving various purposes such as cancer vaccines, blocking or inhibiting agents, and inducers of cell death, among others (192). As this review focuses on immunotherapies that induce ICD, we aimed to describe immunotherapeutic peptides that are capable of inducing ICD.

##### 4.5.2.2 Host defence peptides

An important source of ICD-inducing peptides are the host defence peptides (HDP), also known as antimicrobial peptides (AMPs), which are a conserved component of the innate immune system of a wide range of organisms (193), and they have specific physicochemical properties, such as a net positive charge and a specific distribution of cationic and hydrophobic amino acids (194), which enable their electrostatic interaction with cell membranes, membrane proteins or intracellular targets to promote cell lysis or regulated cell death.

Host defence peptides have immunomodulatory properties, such as modulation of inflammatory responses, chemokine expression, activation and differentiation of leukocytes, stimulation of antigen presentation, among others (193). Many HDP also have antitumour activities, most of them related to overcoming the immunosuppressive microenvironment, including: reduction of immunosuppressive cells, migration of phagocytic cells, reduction of pro-tumour molecules, recruitment of antitumour cytotoxic cells, among others (193). Among the HDP, some have been reported as ICD inducers, such as LTX-315 which, in addition to its effect in reducing pro-tumour immune cells, has also been reported to induce the emission of DAMPs (calreticulin, HMGB1 and ATP) and to induce *in vivo* myeloid and T lymphocyte tumour infiltration (195). The oncolytic peptides DTT-205 and DTT-304 induced calreticulin exposure and HMGB1 release, promoting tumour remission and the development of long-term immune memory against sarcoma and lung cancer cells *in vivo* (196). In addition, the peptide LTX-401 induced the release of ATP and HMGB1, and induced tumour remission with abscopal effect and promoted the establishment of antitumour memory against hepatocellular carcinoma cells *in vivo* (197). Taken together, the diverse effects of HDP could enhance their ICD properties to promote the antitumour immune system activation.

##### 4.5.2.3 Thrombospondin-1 peptide mimics

Thrombospondin-1 (TSP-1) mimic peptides are synthetic sequences (natural or modified) designed to mimic the functions of the different motifs in the TSP-1. In this sense, two sequences with a VVM motif were identified within the C-terminal cell-binding domain (CBD) of TSP-1 (198, 199). This led to the generation of 7N3 (1102-FIRVVMYEGKK-1112) and 4N1 (1016-RFYVVMWK-1024) peptides. Then, a modified version of 4N1, called 4N1K (K-RFYVVMWK-K) was developed, containing two lysine (K) residues flanking the 4N1 sequence, to increase the peptide solubility (198, 200).

4N1K was found to induce cell death in leukemic cells in addition to the modulation of cytokines in DCs and microglial cells (201–204). Thus, to improve these effects a structure-activity relationship study led to the synthesis of the first serum-stable analogue of 4N1K, called PKHB1. PKHB1 is recognized as an ICD inducer in leukemic and breast cancer cells. This peptide induces a calcium-dependent and caspase-independent cell death mechanism. Furthermore, PKHB1-induced cell death exhibits key molecular hallmarks of ICD, including the exposure of calreticulin, HSP70, HSP90, and the release of ATP and HMGB1 (**Figure 4**) in various leukemic and breast cancer cell lines (205–207). Furthermore, PKHB1-treated cells promote DC maturation and stimulate the antitumour response of T-cells *ex vivo*. In a prophylactic context, PKHB1-treated cells prevent the tumour establishment in leukemic and breast cancer tumour bearing mice. PKHB1 also induce tumour shrinkage, increasing cytotoxic T-cell counts in blood and tumours, while reducing MDSCs and regulatory T-cells (T-regs) in breast cancer tumour-bearing mice (207). Notably, PKHB1 possess antiviral properties by triggering ICD in cases of infectious corneal disease caused by Herpes simplex virus type II. This event triggers an antiviral immune response, that reduces viral levels and mitigates the severity of the infection (208). Finally, PKHB1 also promotes the elimination of inflammatory macrophages in models of subretinal and peritoneal inflammation (209). Despite the evaluation of the immunogenicity and the antitumor effect of PKHB1, its impact on innate immune system cells and their role in the antitumor activity of PKHB1 have not been evaluated to date.

##### 4.5.2.4 Other peptides as ICD-inductors

F-pY-T is a mitochondria-targeting peptide that has been reported as an ICD inducer, triggering calreticulin exposure (*in vitro* and *in vivo*), ATP and HMGB1 release. F-pY-T *in vivo* induced DC maturation and promoted the intratumoral infiltration of CD8+ cells, and inhibited tumour growth (210).

The recombinant human milk peptide lactaptin RL2 induced calreticulin exposure, ATP and HMGB1 release in breast cancer cells and promoted the phagocytosis of dying-cancer cells by macrophages. *In vivo*, vaccination with RL2-treated cells also increased the survival of mice (211).

The calmodulin binding peptide CBP501 has been reported as an ICD inducer, which promotes calreticulin exposure and HMGB1 release, and increases *in vivo* mouse survival in vaccination experiments (in combination with cisplatin).Also, the combination of cisplatin and CBP501 also reduces tumour growth and increases intratumoral CD8+ cell infiltration (212).

Other peptides that have been demonstrated to possess immunomodulatory proprieties and induce immunogenic cell death are peptide-based proteasome inhibitors. Proteasome inhibitors are a class of drugs whose main mechanism is to inhibit the multi-protease subunits of the proteosome, leading to the accumulation of undegraded proteins, affecting different cellular processes which lead to cell death (213). Bortezomib is a dipeptide boronic acid derivative that acts as a reversible inhibitor of the 26S proteasome and is the first FDA approved proteosome inhibitor (214). It has been shown to have various immunomodulatory effects in allogeneic stem cell transplantation, antibody-mediated graft rejection and various inflammatory diseases (215). Furthermore, it is considered an ICD inducer, as it (1) prevents breast cancer tumour establishment (216) (2); promotes HSP90 exposure, DC maturation and antitumour T-cell response against myeloma cells from patients (217) (3); triggers calreticulin exposure, induces DCs maturation (increase of CD83 and CD86) and the antitumour T-cell response, increasing the number of effector memory CD4+ and CD8+ cells (4); the *in vivo* vaccination with bortezomib-treated cells prevents tumour establishment and promotes long-term antitumour memory against multiple myeloma cells (218).

## 5 Discussion and concluding remarks

Since 1891, when Coley used the first immunotherapies, there have been tremendous advances and discoveries that have revealed the promising potential of immunotherapies for the prevention and treatment of cancer. Although these are usually combined with other cancer treatments capable of killing cancer cells to attack cancer cells from different perspectives, some immunotherapies have the capacity to be cytotoxic to cancer cells through immunogenic cell death induction. Although few in number, these ICD-inducing immunotherapies represent a promising and innovative approach in the fight against cancer, with the innate immune system playing a key role in their success.

ICD has the potential to induce a robust antitumor immune response (219). However, the principal challenge is associated with treatment resistance, which could hamper its therapeutic efficacy. This may be related with the ICD induction which depends on the host (for example immune perception of ICD), the tumour (for example DAMPs’ exposure), the ICD inductor (for example, its immunosuppressive effects), or the specific cancer microenvironment (the specific immunosuppressive cells present in the TME) (220). Cell death resistance could be addressed by combination regimens of therapeutic alternatives that could attack from different sources. For example, it has been demonstrated that bortezomib improves adoptive T cell therapy by sensitizing cancer cells to FasL cytotoxicity (221). Also, oncolytic viruses provide potent antitumor effects against brain tumours when combined with adoptive T-cell therapy (222). While, bovine dialyzable leukocyte extract, which induces ICD in breast cancer, when combined with cyclophosphamide induces synergic cell death (223). The combination of chemotherapy with immunotherapies is a primary approach evaluated to overcome cancer cell resistance (224–226). However, these combinations mostly look for ICD inducing chemotherapies with non-necessarily ICD inducing immunotherapies, yet combination of different ICD-inducing agents might promote better responses. Especially if combining immunotherapeutic and chemotherapeutic agents that possess ICD potential and immunomodulatory properties, as it has also been described for certain types of chemotherapies (227, 228). Immunotherapies may help to surpass tumour resistance mechanisms, as immunotherapies that stimulate the innate immune system may augment ICD by enhancing the effect triggered by DAMPs, thereby promoting a robust immune response (229). Additionally, they may activate DCs, enhancing antigen presentation and promoting the recruitment and activation of effector immune cells (125), like NK cells, which in turn can efficiently trigger cellular cytotoxicity, potentially leading to ICD (101). This synergy is a promising alternative to overcome ICD resistance, providing an interesting avenue for enhanced antitumor effects and improved therapeutic outcomes.

**Figure 5:**
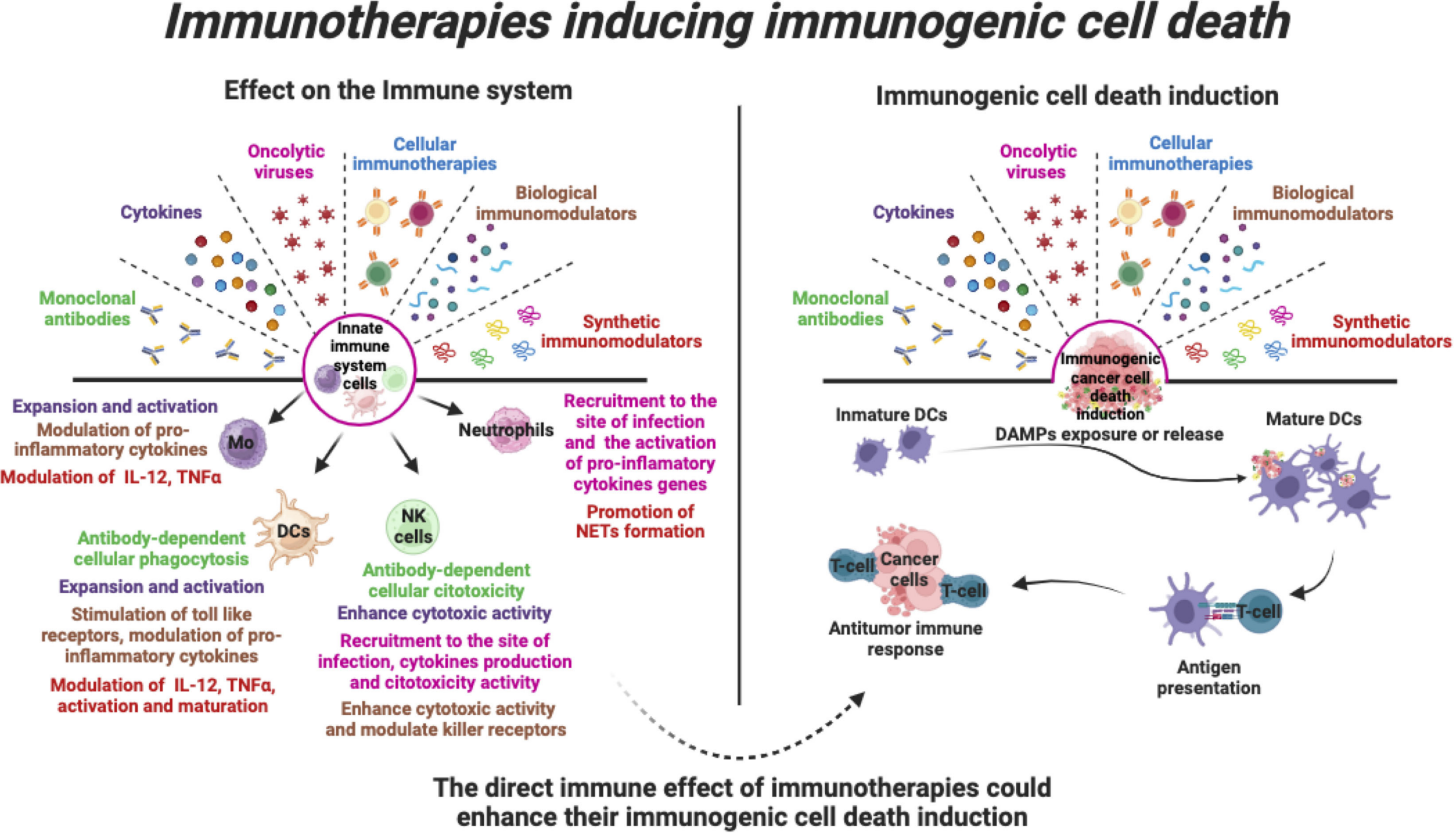
Immunotherapies that induce immunogenic cell death. Various immunotherapies promote the stimulation of various pro-inflammatory responses in cells of the innate immune system. In addition, immunotherapies can induce direct cell death of tumour cells through induction of immunogenic cell death, leading to activation of the anti-tumour immune system. Thus, the direct effect of immunotherapies on innate immune cells could enhance their ICD induction and enhance the anti-tumour immune response.

The intricate interplay between ICD and the innate immune response opens new avenues for the development of more effective and durable cancer treatments with promising potential. These onco-immunotherapies, including monoclonal antibodies, cytokines, oncolytic viruses, cellular immunotherapies, and other biological or synthetic immunomodulators, have clearly demonstrated their potential to harness the body’s natural defences against cancer cells. By triggering the ICD, these treatments also facilitate the release of tumour antigens and danger signals, stimulating innate immune cells such as dendritic cells, natural killer cells and macrophages. Activation of these innate first-line defence cells is critical for mounting a potent and sustained anti-cancer response, which involves the durable long-term memory of the adaptive immune system (**Figure 5**).

As these potential actions of immunotherapies have not been the primary focus, several challenges remain. For example, uncovering the potential role of immunotherapies in inducing immunogenic cell death is a significant challenge, given that only a few of them have been studied as ICD inducers. This is particularly striking when compared to the large body of evidence highlighting their role in the immune system. Another aspect is not only to elucidate their role in ICD induction, but also to propose combinations that enhance this dual action for a more comprehensive approach against a wide range of cancers. In conclusion, we recommend that these strategies be emphasised, as addressing these aspects will undoubtedly contribute to a deeper understanding, more effective use and further development of the enormous potential offered by these immunotherapies.

## Author contributions

KC-R: Data curation, Investigation, Methodology, Validation, Writing – original draft, Writing – review and editing. HL-A: Data curation, Investigation, Methodology, Writing – original draft, Writing – review and editing. CR-P: Writing – review and editing. AM-T: Conceptualization, Data curation, Investigation, Methodology, Validation, Writing – original draft, Writing – review and editing. DS-A: Conceptualization, Validation, Writing – review and editing.

## Glossary

**Table:**

ICD	Immunogenic cell death
DAMPs	Damage-associated molecular patterns
PRRs	Pattern recognition receptors
TAAs	Tumour-associated antigens
MHC	Major histocompatibility complex
TME	Tumour microenvironment
PAMPs	Pathogen-associated molecular patterns
DCs	Dendritic cells
mAb	Monoclonal antibody
ADCC	Antibody-dependent cellular cytotoxicity
CDC	Complement-dependent cytotoxicity
ADCP	Antibody-dependent cellular phagocytosis
NK	Natural killer
BCMA	B-cell maturation antigen
CRT	Calreticulin
HSP	Heat shock protein
ATP	Adenosine triphosphate
HMGB1	high mobility group box protein 1
FOLFIRI	5-fluorouracil, irinotecan and leucovorin
ER	Endoplasmic reticulum
TAMs	Tumour associated macrophages
PDT	Photodynamic therapy
TNF-α	Tumour necrosis factor alpha
IFN-γ	Interferon gamma
FDA	Food and Drug Administration
GM-CSF	Granulocyte macrophage colony-stimulating factor
G-CSF	Granulocyte-colony stimulating factor
SMAC	Secondary mitochondria-derived activator of caspases
PANoptosis	Pyroptosis apoptosis and necroptosis cell death
OVs	Oncolytic viruses
NDV	Newcastle disease virus
TLRs	toll like receptors
APCs	antigen presenting cells
oHSV	herpes simplex virus
TGFβ	transforming growth factor beta
PDAC	Human Pancreatic ductal adenocarcinoma cells
TMD	tumour micro-vessel density
CAR	chimeric antigen receptor
CRS	cytokine release storms
GvHD	graft versus host disease
MDSCs	myeloid-derived suppressor cells
CAR M	CAR macrophages
NO	nitric oxide
ROS	reactive oxygen species
BCG	Bacille-Calmette-Guerin
MCP-1	Monocyte chemoattractant protein-1
UC	urothelial carcinoma
DLE	dialysable leukocyte extracts
I-CRP	Immunepotent CRP
bDLE	dialysable leukocyte extract obtained from bovine spleen
LPS	Lipopolysaccharide
IMQ	Imiquimod
TLR7	toll like receptor 7
PBMC	peripheral blood mononuclear cells
IDO	indoleamine 2, 3-dioxygenase
HDP	host defence peptides
AMPs	antimicrobial peptides
TSP-1	Thrombospondin 1
CBD	C-terminal cell-binding domain
T-regs	regulatory T-cells
